# Supplementary material for: Serum Urate Levels and Ultrasound Characteristics of Carotid Atherosclerosis across Obesity Phenotypes
Source: Biomedicines. 2023 Jul 4;11(7):1897. doi: 10.3390/biomedicines11071897 (PMC10376805; doi:10.3390/biomedicines11071897)
Supplement: Supplementary file 1 [file biomedicines-11-01897-s001.zip › biomedicines-2464550-supplementary.pdf]

Supplementary Material

Serum urate levels and ultrasound characteristics of carotid atherosclerosis accross obesity phenotypes

Daniela Efremova[1], Natalia Ciobanu[1], Danu Glavan[1], Pavel Leahu[1,2], Renata Racila[1,2], Tatiana Bălănuță[1], Alexandru Matei[1], Maria Vasilieva[1,2], Cristina Chepte[1], Paula Bîtcă[1], Cristina Damian[1], Ana Bondarciuc[1], Irina Bejenari[1], Adelina Cojocaru[1], Diana Manea[1], Mihail Ciocanu[1], Eremei Zota[1], Dumitru Ciolac[1,2*], Stanislav Groppa[1,2*]

[1] Department of Neurology, Institute of Emergency Medicine, 2004 Chisinau, Republic of Moldova

[2] Department of Neurology, Nicolae Testemițanu State University of Medicine and Pharmacy, 2004 Chisinau, Moldova

***Corresponding authors**:
Stanislav A. Groppa, MD, PhD, Prof.
Department of Neurology, Institute of Emergency Medicine, Toma Ciorba street 1, MD-2004, Chisinau, Republic of Moldova; Tel: + 373-22-205312; Fax: + 373-22-235300
Email: stanislav.groppa@usmf.md

Dumitru Ciolac, MD, PhD
Department of Neurology, Institute of Emergency Medicine, Toma Ciorba street 1, MD-2004, Chisinau, Republic of Moldova; Tel: + 373-22-925781
Email: dimaciolac@gmail.com

Supplementary Figures

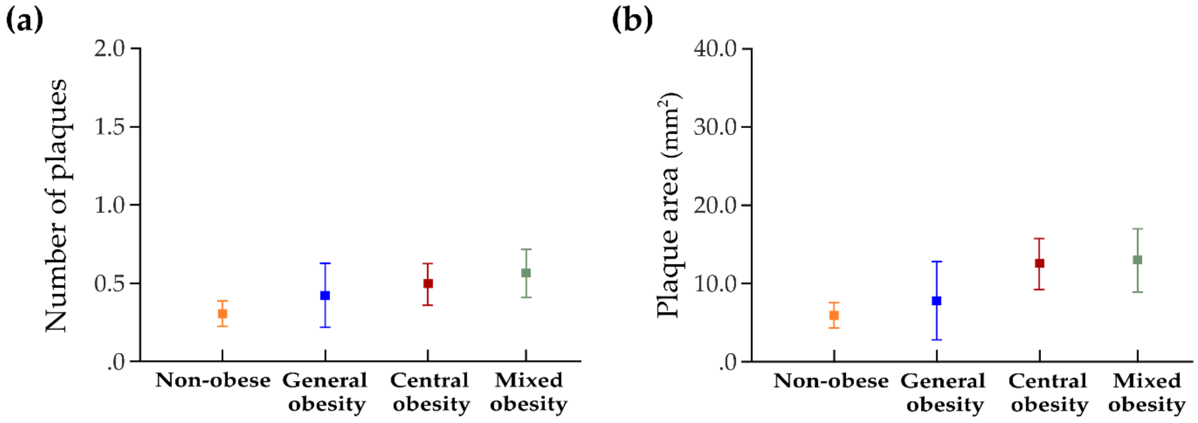

Figure S1. Number and area of plaques across subject groups. Comparison of number of the plaques (a) and total area of the plaque (b) between non-obese, subjects with general, central and mixed obesity. Error bars with 95% confidence interval for the mean are shown.

(a)

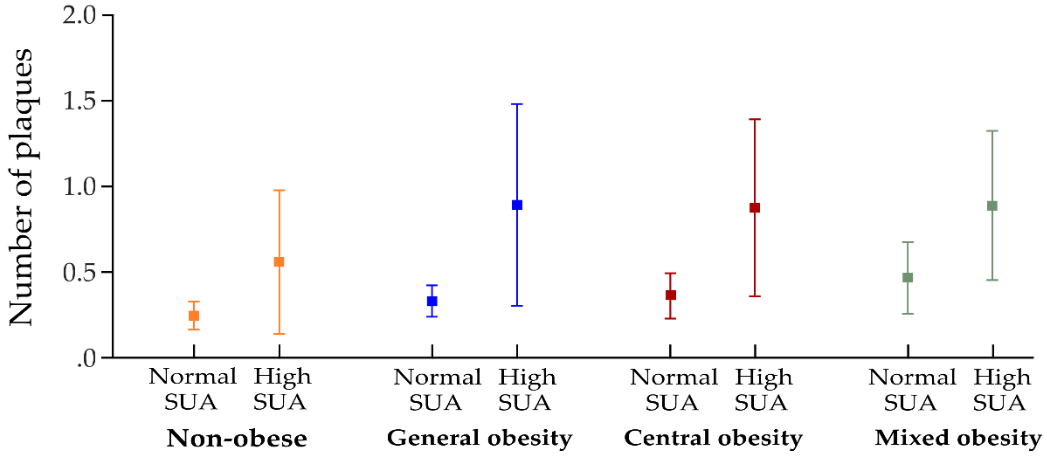

(b)

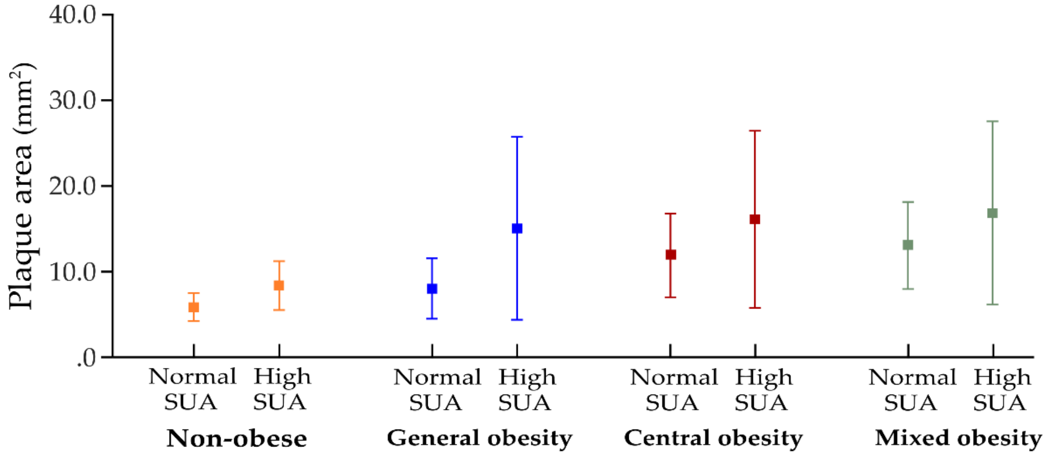

Figure S2. Subgroup analysis of number and area of plaques. Comparison of number of the plaques (a) and total area of the plaque (b) between subjects with normal and high serum uric acid (SUA) levels among non-obese, general, central and mixed obesity groups. Error bars with 95% confidence interval for the mean are shown.
